# Rolle, Arbeitsweise und Empfehlungen der Ständigen Impfkommission (STIKO) im Kontext der COVID-19-Pandemie

**DOI:** 10.1007/s00103-022-03610-2

**Published:** 2022-11-22

**Authors:** Sabine Vygen-Bonnet, Johanna Schlaberg, Judith Koch

**Affiliations:** Fachgebiet Impfprävention, Robert Koch-Institut, Seestr. 10, 13353 Berlin, Deutschland

**Keywords:** COVID-19, SARS-CoV‑2, STIKO, Impfkommission, Empfehlung, Pandemie, Evidenzbasierte Medizin, EBM, Impfung, COVID-19, SARS-CoV‑2, STIKO, NITAC, Recommendation, Pandemic, Evidence-based medicine, EBM, Immunization

## Abstract

Die Ständige Impfkommission (STIKO) ist ein ehrenamtliches Gremium, dessen 18 ExpertInnen vom Bundesministerium für Gesundheit (BMG) berufen werden. Die wissenschaftliche Arbeit der STIKO wird durch eine Geschäftsstelle am Robert Koch-Institut (RKI) unterstützt. Die STIKO erarbeitet unabhängige Impfempfehlungen für Deutschland mit der Methodik der evidenzbasierten Medizin (EBM).

Während der COVID-19-Pandemie sah sich die STIKO mit großen Herausforderungen konfrontiert. Innerhalb kürzester Zeit wurden mehrere COVID-19-Impfstoffe z. T. neuer Technologien zugelassen. Die Nutzen-Risiko-Abwägung wurde nach dem jeweils aktuellen Wissensstand durchgeführt. Die Impfempfehlungen mussten fortlaufend angepasst werden an die sich stetig ändernde Epidemiologie von SARS-CoV‑2, zunehmende Impfstoffverfügbarkeiten, neue Zulassungen, Indikationserweiterungen und neue Sicherheitssignale (z. B. Sinusvenenthrombosen nach Vektor-basierten Impfstoffen). Die STIKO hat ihre Arbeitsweise der Situation angepasst und während der Pandemie Beeindruckendes geleistet. Sie hat auch unter Zeitdruck die EBM-Prinzipien beachtet und Impfempfehlungen basierend auf der jeweils verfügbaren Evidenz erarbeitet. Jede Impfempfehlung wurde vor der endgültigen Beschlussfassung in ein Stellungnahmeverfahren mit den betroffenen Fachkreisen (z. B. med. Fachgesellschaften, Gesundheitsbehörden) gegeben. Dabei wurde trotz der kurzen Fristen umfassend und konstruktiv kommentiert und der STIKO die Möglichkeit gegeben, ihre Empfehlungen unter Berücksichtigung der Stellungnahmen zu diskutieren, anzupassen und somit auf breiten Konsens zu bauen.

Die zurückliegenden Monate haben gezeigt, dass es möglich und sinnvoll ist, Impfempfehlungen auch während einer Pandemie nach den Prinzipien der EBM zu erarbeiten. Ausreichende personelle Ressourcen in der STIKO-Geschäftsstelle sind dabei essentiell.

## Abstract (en)

The Standing Committee on Vaccination (STIKO) is a voluntary body whose 18 experts are appointed by the Federal Ministry of Health. The scientific work of STIKO is supported by a scientific secretariat at the Robert Koch Institute. The STIKO develops independent vaccination recommendations for Germany using the methodology of evidence-based medicine (EBM).

During the COVID-19 pandemic, STIKO faced major challenges. Several COVID-19 vaccines based on new technologies were approved within a very short time. The benefit–risk assessment had to be carried out according to the current state of knowledge. The vaccination recommendations had to be continuously adapted to the constantly changing epidemiology of SARS-CoV‑2, increasing vaccine availability, new approvals, extensions of indications, and safety signals of vaccines. STIKO has adapted its way of working to the situation; the experts showed an impressive commitment during the pandemic. Even under time pressure, STIKO adhered to the principles of EBM and developed evidence-based vaccination recommendations. Before the final decision was made, STIKO submitted every vaccination recommendation to a commenting procedure with the stakeholders (e.g., medical societies and health authorities). Despite the short deadlines, the stakeholders made extensive and constructive comments and gave STIKO the opportunity to discuss and adapt their recommendations, consider the feedback, and thus build on a broad consensus.

The past few months have shown that it is possible and rational to developing vaccination recommendations based on the principles of EBM even during a pandemic. Sufficient human resources in the STIKO office are essential.

## Einleitung

Die Ständige Impfkommission (STIKO) entwickelt für die Bevölkerung Deutschlands Impfempfehlungen, die sich auf in der Europäischen Union (EU) zugelassene Impfstoffe beziehen. Die STIKO erarbeitet ihre Empfehlung mit Methoden der evidenzbasierten Medizin (EBM). Sie prüft dabei nicht nur den Nutzen der Impfung für das geimpfte Individuum, sondern berücksichtigt auch die potenziellen Effekte der Impfung auf die gesamte Bevölkerung und bezieht weitere Aspekte, wie die Umsetzbarkeit der Impfempfehlung und die Akzeptanz der Impfung in der Bevölkerung, in ihre Bewertung mit ein. Neben Empfehlungen zu Standard- und Indikationsimpfungen gibt die STIKO Empfehlungen für Impfungen im Rahmen von Ausbruchsgeschehen bzw. Pandemien. Hier kommt dem Impfen eine besondere Bedeutung für den Schutz der bisher nicht infizierten Bevölkerung vor Erkrankung und für die Eindämmung der Weiterverbreitung des Erregers zu.

Die Impfkampagne im Rahmen der Coronavirus-Disease-2019(COVID-19)-Pandemie ist im Vergleich zu in der Vergangenheit aufgebauten Impfprogrammen in vieler Hinsicht einzigartig: Noch nie wurden Impfstoffe gegen einen neuen Erreger und mit einer neuen Impfstofftechnologie so schnell – im Zeitraum von weniger als einem Jahr – entwickelt, in klinischen Studien geprüft und für den Einsatz in der Bevölkerung zugelassen. Zu Beginn der Pandemie wurde an mehr als 170 Impfstoffkandidaten geforscht und weitere 15 Impfstoffe befanden sich in der klinischen Erprobung. Noch nie wurde in Deutschland in nur wenigen Monaten ein so großer Anteil der Bevölkerung geimpft. Noch nie war daher die STIKO gefordert, Impfempfehlungen innerhalb kürzester Zeit zu erstellen und über Monate hinweg fortlaufend zu aktualisieren und an die sich verändernde Epidemiologie und neue Erkenntnisse anzupassen.

Die Ständige Impfkommission konstituierte sich 1972 und besteht im Jahr 2022 seit 50 Jahren. Die STIKO ist beim Robert Koch-Institut (RKI) angesiedelt, wo sich auch ihre Geschäftsstelle befindet [1]. Die Aufgaben der Kommission sind im Infektionsschutzgesetz (IfSG) seit 2001 gesetzlich festgelegt. Nach § 20 Abs. 2 gibt die Kommission Empfehlungen zur Durchführung von Schutzimpfungen und anderer Maßnahmen der spezifischen Prophylaxe, auf deren Grundlage die obersten Landesgesundheitsbehörden der Bundesländer ihre Empfehlungen aussprechen [2]. Der Gemeinsame Bundesausschuss (G-BA) entscheidet seit 2007 auf Basis der STIKO-Empfehlungen, ob eine Impfung in die Schutzimpfungsrichtlinie aufgenommen und zur Pflichtleistung der Gesetzlichen Krankenversicherung wird.

Die STIKO hat bis zu 18 ehrenamtlich tätige Mitglieder. Die ExpertInnen kommen aus verschiedenen Fachdisziplinen (z. B. Pädiatrie, Virologie, Allgemeinmedizin, Immunologie, Epidemiologie, Arbeitsmedizin, Öffentlicher Gesundheitsdienst) und werden alle 3 Jahre vom Bundesministerium für Gesundheit (BMG) neu berufen. Um die Transparenz der Kommissionsarbeit zu stärken, werden die Mitglieder seit mehr als 10 Jahren aufgefordert, vor ihrer Berufung und vor jeder regulären STIKO-Sitzung mögliche Interessenkonflikte in einem standardisierten Fragebogen offenzulegen. Mitglieder, bei denen zu einzelnen Beratungsgegenständen ein Anschein von Befangenheit besteht, z. B. weil sie an einer Impfstoffstudie zu der betreffenden Erkrankung aktiv oder beratend beteiligt sind, dürfen bei der Beratung und Beschlussfassung nicht mitwirken.

Die STIKO trifft sich regulär 3‑mal im Jahr in Präsenz, um über neue oder angepasste Impfempfehlungen zu beraten. Seit 2011 werden die Impfempfehlungen nach der Standardvorgehensweise (SOP) für die Entwicklung evidenzbasierter Impfempfehlungen erarbeitet [3]. Diese beinhaltet die systematische Aufarbeitung aller relevanten Inhalte anhand eines vorgegebenen Fragenkatalogs durch die wissenschaftlichen MitarbeiterInnen der Geschäftsstelle der STIKO. Die WissenschaftlerInnen arbeiten dabei eng mit den STIKO-Mitgliedern in Arbeitsgruppen zu den einzelnen Impfungen bzw. zu den impfpräventablen Erkrankungen zusammen. Mittels mathematischer Modellierungen, die durch Mitarbeitende des Fachgebietes Impfprävention des RKI oder externe Partner durchgeführt werden, werden direkte und indirekte Effekte einer neuen oder geänderten Impfempfehlung anhand von unterschiedlichen Szenarien abgeschätzt. Die Ergebnisse dieser Berechnungen sind ein wichtiger Baustein im Entscheidungsprozess und in der abschließenden Nutzen-Risiko-Abwägung der STIKO.

Bevor eine Impfempfehlung verabschiedet wird, geht der Entwurf zusammen mit der wissenschaftlichen Begründung üblicherweise in ein 6‑wöchiges Stellungnahmeverfahren. Eine neue Empfehlung wird durch die Veröffentlichung im Epidemiologischen Bulletin gültig und erscheint zusammen mit der wissenschaftlichen Begründung. Für die Begleitkommunikation (Pressemitteilungen, Faktenblätter, Impfnews in der STIKO-App, „Antworten auf häufig gestellte Fragen“ (FAQs)) wird die STIKO von einem Kommunikationsteam des Fachgebiets Impfprävention am RKI unterstützt. Da die Ressourcen in der Geschäftsstelle sowie in der STIKO begrenzt sind, werden zu Beginn der Berufungsperiode die Themen des Arbeitsplans durch die Kommission nach ihrer Wichtigkeit und Dringlichkeit priorisiert und das zeitliche Vorgehen abgestimmt.

Die beschriebene Vorgehensweise hat auch während einer Pandemie unabhängig vom auslösenden Erreger Bestand. Auch im Rahmen der COVID-19-Pandemie hat die STIKO die gesetzliche Aufgabe, auf Basis der verfügbaren Daten zur Epidemiologie der Erkrankung und zu den verfügbaren Impfstoffen evidenzbasierte Impfempfehlungen für die deutsche Bevölkerung zu entwickeln und diese immer wieder dem aktuellen Wissensstand anzupassen. Bei limitierten Impfstoffressourcen bilden Risikogruppen-spezifische Empfehlungen der STIKO die Grundlage für die Impfstoffverteilung.

## Arbeit der STIKO während der COVID-19-Pandemie

Das Severe Acute Respiratory Syndrome Coronavirus Type 2 (SARS-CoV-2), der Erreger von COVID-19, breitete sich nach dem ersten Auftreten im Dezember 2019 weltweit aus. Im März 2020 wurde das Ausbruchsgeschehen von der Weltgesundheitsorganisation (WHO) zur Pandemie erklärt. Im Rahmen der COVID-19-Pandemie sind bis zum 22.06.2022 weltweit 540 Mio. COVID-19-Erkrankungen und 6,3 Mio. Todesfälle registriert worden [4]. In Deutschland sind in diesem Zeitraum 27,5 Mio. SARS-CoV-2-Fälle und > 140.000 COVID-19-Todesfälle übermittelt worden. Die STIKO hat im Juni 2020 eine COVID-19-Arbeitsgruppe unter Einbeziehung einer Expertin aus dem Paul-Ehrlich-Institut (PEI) und einer Public-Health-Ethikerin gegründet. Am 21.12.2020 wurde der erste COVID-19-Impfstoff Comirnaty von BioNTech/Pfizer in der EU zugelassen (Tab. 1). Die STIKO hat ihre erste COVID-19-Impfempfehlung und die zugehörige wissenschaftliche Begründung am 17.12.2020, kurz vor Beginn der Verfügbarkeit des ersten COVID-19-Impfstoffes in Deutschland veröffentlicht. Gegenstand dieser Impfempfehlung waren die Daten zur Sicherheit und Wirksamkeit von Comirnaty bei Personen im Alter von ≥ 16 Jahren und die Priorisierung von Personengruppen, denen als Erstes eine Impfung angeboten werden sollte.

**Table 1:** 

Name (Hersteller)	Impfstofftyp	Altersgruppe	Dosierung	Grundimmunisierung (GI)	Empfohlen zur Auffrischimpfung	Besonderheiten
*Comirnaty (BioNTech/Pfizer)*	mRNA	5–11 Jahre	10 µg	1 bzw. 2 Dosen^a^	Ja, bei Kindern mit Vorerkrankungen und Immundefizienz	–
		≥ 12 Jahre	30 µg		Ja	Seltene unerwünschte Ereignisse: Peri‑/Myokarditis
*Spikevax (Moderna)*	mRNA	Zugelassen für die Altersgruppe 6–11 Jahre	50 µg	1 bzw. 2 Dosen^a^	Ja, bei Kindern mit Vorerkrankungen und Immundefizienz	Sicherheitsdaten bisher nur aus Zulassungsstudien, daher wird präferenziell Comirnaty empfohlen
		≥ 30 Jahre	GI: 100 µg; AI Immungesunde: 50 µg; AI bei Immundefizienz: 100 µg		Ja, bei Erwachsenen ≥ 30 Jahren	Seltene unerwünschte Ereignisse: Peri‑/Myokarditis; Myokarditisrisiko bei ≥ 12- bis < 30-Jährigen erhöht, daher nicht empfohlen in dieser Altersgruppe
*Vaxzevria (AstraZeneca)*	Vektor-basiert	Seit 01.12.2021 in Deutschland nicht mehr verfügbar	≥ 2,5 × 10^8^ IE	2 Dosen	Nein	Aufgrund seltener thromboembolischer Ereignisse Altersbeschränkung auf ≥ 60 Jahre
*JCOVDEN, vormals COVID-19 Vaccine Janssen (Janssen Cilag International)*	Vektor-basiert	≥ 60 Jahre	≥ 8,92 log_10_ IE	Zugelassen als Einzeldosis; Optimierung mit einer mRNA- oder Nuvaxovid-Impfstoffdosis empfohlen	Nein	Aufgrund ungenügender Effektivität Optimierung der GI empfohlen; aufgrund seltener thromboembolischer Ereignisse Altersbeschränkung auf ≥ 60 Jahre
*Nuvaxovid (Novavax)*	Adjuvantierter Proteinimpfstoff	≥ 18 Jahre	5 µg	2 Dosen	Nein	Anwendung während der Schwangerschaft und Stillzeit wird aufgrund fehlender Daten zur Wirksamkeit und Sicherheit des enthaltenen Adjuvans Matrix M derzeit nicht empfohlen, kann jedoch in Einzelfällen erwogen werden, z. B. wenn eine produktspezifische, medizinische oder sonstige Kontraindikation gegen mRNA-Impfstoffe besteht

*AI* Auffrischimpfung, *D* Deutschland, *GI* Grundimmunisierung, *IE* infektiöse Einheiten

^a^1 Impfstoffdosis für gesunde Kinder, 2 Impfstoffdosen für Kinder mit Vorerkrankungen inkl. Immundefizienz und für gesunde Kinder, in deren Umfeld sich Angehörige oder andere Kontaktpersonen mit hohem Risiko für einen schweren COVID-19-Verlauf befinden, die durch eine Impfung selbst nicht sicher geschützt werden können

Gemeinsam mit der Leopoldina und dem deutschen Ethikrat hat die STIKO einen Priorisierungsplan erarbeitet, der empfahl, wie die initial begrenzt verfügbaren Impfstoffe verteilt werden sollten [5]. Dieser Plan sollte den für die Durchführung der Impfungen verantwortlichen Stellen, aber auch der STIKO für alle folgenden Impfempfehlungen als Grundlage dienen. Ziel war es, möglichst viele Todesfälle zu verhindern und die Arbeitsfähigkeit des Gesundheitssystems zu erhalten.

Um Vorerkrankungen mit einem erhöhten Risiko zu identifizieren, führte die COVID-19-Arbeitsgruppe der STIKO zusammen mit Mitarbeitenden der Geschäftsstelle sowie dem Fachgebiet Impfprävention am RKI einen sogenannten Umbrella-Review zu prädisponierenden Faktoren für einen schweren COVID-19-Verlauf durch [6]. Dafür wurden 52 systematische Übersichtsarbeiten und die darin enthaltenen 194 Primärstudien analysiert und bewertet. Daneben wurden zahlreiche Publikationen zum Altersrisiko für einen schweren Erkrankungsverlauf und zu tätigkeits- und berufsbedingten Expositionsrisiken berücksichtigt. Die Mehrzahl der Studien war zum Zeitpunkt der Beurteilung noch nicht in einem Peer-Review-Verfahren begutachtet worden.

Entsprechend der von der Kommission erarbeiteten Priorisierung, die die einzelnen Bevölkerungsgruppen nach ihrem Risiko für schwere COVID-19-Verläufe bzw. nach ihrem tätigkeitsbedingten Expositionsrisiko einer der 6 Priorisierungsstufen zuordnete, wurde am 26.12.2020 das COVID-19-Impfprogramm in Deutschland gestartet. Es begann mit Personen im Alter von ≥ 80 Jahren, BewohnerInnen von Senioren- und Altenpflegeheimen, Personal mit besonders hohem Expositionsrisiko in medizinischen Einrichtungen, Personal in medizinischen Einrichtungen mit engem Kontakt zu vulnerablen Gruppen (z. B. Onkologie und Transplantationsmedizin), Pflegepersonal in der ambulanten und stationären Altenpflege und anderen Tätigen in Senioren- und Altenpflegeheimen mit Kontakt zu den BewohnerInnen.

**Figure 1:**
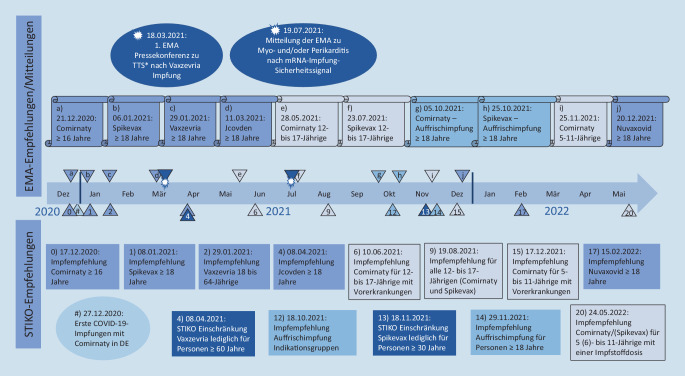

Zur Beratung der ersten Impfempfehlung fanden 7 Treffen der COVID-19-Arbeitsgruppe der STIKO und 4 Beratungen mit der gesamten STIKO statt. Bis Mai 2022 wurden insgesamt 20 Aktualisierungen der COVID-19-Impfempfehlung (Tab. 2; Abb. 1) in 3 regulären STIKO-Sitzungen und 47 zusätzlichen, ausschließlich COVID-19 gewidmeten Online-Beratungen erarbeitet. In der Abb. 1 sind für die Zeit seit dem Beginn der COVID-19-Impfkampagne in Deutschland besonders gesundheitsrelevante Empfehlungen der Europäischen Arzneimittel-Agentur (EMA) sowie die darauf bezugnehmenden Empfehlungen der STIKO dargestellt. Dies soll verdeutlichen, in welchem engen zeitlichen Abstand die Empfehlungen der STIKO auf die Empfehlungen der EMA sowie die zugehörige Zulassung der EU-Kommission in den meisten Fällen folgten. Die zugrunde liegende Evidenzaufarbeitung wurde von Mitarbeitenden der STIKO-Geschäftsstelle und des Fachgebiets Impfprävention sowie von STIKO-Mitgliedern durchgeführt. Zusätzlich fanden zahlreiche Treffen von Unterarbeitsgruppen der STIKO statt, die unter Beteiligung externer ExpertInnen zu spezifischen COVID-19-Impfempfehlungsthemen wie Schwangerschaft und Stillzeit, Kinder und Jugendliche, Personen mit Immundefizienz, Impfnebenwirkungen und Impfung von Genesenen berieten. Während der gesamten Zeit haben Mitarbeitende des PEI die STIKO fortlaufend über den Stand der Zulassungen neuer Impfstoffe und von Zulassungsanpassungen sowie zu Ergebnissen aus der Surveillance von unerwünschten Wirkungen informiert.

**Table 2:** 

Publikation	Datum	Inhalt/Neuerungen
COVID-19-Empfehlung [5]	14.01.2021, online vorab am 17.12.2020	**Comirnaty** für Jugendliche ab 16 Jahren und Erwachsene
		Priorisierungsempfehlung
*1. Aktualisierung* [21]	14.01.2021, online vorab am 08.01.2021	**Spikevax** für Erwachsene ≥ 18 Jahre
*2. Aktualisierung* [7]	04.02.2021, online vorab am 29.01.2021	**Vaxzevria** für Personen im Alter von 18 bis 64 Jahren
*3. Aktualisierung* [22]	25.03.2021, online vorab 12.03.2021	**Vaxzevria** für alle Erwachsenen ≥ 18 Jahre Impfung von Genesenen
*4. Aktualisierung* [10]	Beschluss 01.04.2021, wiss. Begründung: 22.04.2021, online vorab 08.04.2021	Einschränkung der **Vaxzevria**-Impfung auf die Altersgruppe **≥** **60 Jahre**
		Empfehlung zum Vorgehen bei bereits einmalig mit **Vaxzevria** Geimpften im Alter < 60 Jahren
		Festlegung des **Impfabstandes** der mRNA-Vakzine auf **6 Wochen**
		Empfehlung des Vektor-basierten 1‑Dosen-**COVID-19-Vaccine-Janssen-Impfstoffs (JCOVDEN)** ≥ 18 Jahre
		Aufnahme von PatientInnen mit chronischen, dialysepflichtigen Nierenerkrankungen in die Priorisierungsstufe 2
*5. Aktualisierung* [12]	12.05.2021, online vorab am 28.04.2021	Einschränkung der **Vaxzevria- und JCOVDEN-Impfung auf Personen im Alter** **≥** **60 Jahre**
*6. Aktualisierung* [23]	10.06.2021	**Comirnaty für 12- bis 17-jährige Kinder und Jugendliche mit Vorerkrankungen **oder für Kinder und Jugendliche, in deren Umfeld sich Kontaktpersonen mit dem Risiko für einen schweren COVID-19-Verlauf befinden, oder aufgrund beruflicher Indikation
*7. Aktualisierung* [24]	24.06.2021	**Aufhebung der Priorisierung**
		Antikörper-Nachweis gleichwertig mit PCR zum Nachweis der Genesung
*8. Aktualisierung* [17]	08.07.2021	**Heterologes Impfschema**
*9. Aktualisierung* [25]	19.08.2021	**Spikevax für 12- bis 17-jährige Kinder und Jugendliche**
		**Allgemeine Impfempfehlung für 12- bis 17-jährige Kinder und Jugendliche**
*10. Aktualisierung* [26]	23.09.2021, online vorab am 17.09.2021	**Schwangere und Stillende**
*11. Aktualisierung* [27, 28]	30.09.2021, online vorab am 24.09.2021	Impfung bei **Immundefizienz ab 12 Jahre**
		**Koadministration **mit Totimpfstoffen
*12. Aktualisierung* [29]	28.10.2021, online vorab am 18.10.2021	**Auffrischimpfung für ≥** **70-Jährige sowie Indikationsgruppen**
		**Optimierung der JCOVDEN-Grundimmunisierung**
		**Berücksichtigung serologischer Nachweis**
*13. Aktualisierung* [16]	18.11.2021	**Spikevax nur für ≥** **30-Jährige**
*14. Aktualisierung* [18]	02.12.2021, online vorab am 29.11.2021	**Auffrischimpfung für alle ≥** **18-Jährigen**
		**Impfung für Genesene**
*15. Aktualisierung* [30]	06.01.2022, online vorab am 17.12.2021	**Kinder 5 bis 11 Jahre mit Vorerkrankungen **oder für Kinder, in deren Umfeld sich Kontaktpersonen mit dem Risiko für einen schweren COVID-19-Verlauf befinden
*16. Aktualisierung* [19]	13.01.2022, online vorab am 21.12.2021	**Auffrischimpfung für ≥** **18-Jährige im Abstand von 3 Monaten zur abgeschlossenen Grundimmunisierung**
*17. Aktualisierung* [31, 32]	20.01.2022	**Auffrischimpfung für 12- bis 17-Jährige mit dem mRNA-Impfstoff Comirnaty (30** **μg), **in einem Zeitfenster von 3 bis 6 Monaten nach der abgeschlossenen Grundimmunisierung
		**Optimierung der Grundimmunisierung mit einem mRNA-Impfstoff nach vorausgegangener Impfung mit JCOVDEN **(3. Impfstoffdosis)
*18. Aktualisierung* [33, 34]	17.02.2022, online vorab am 15.02.2022	**2. Auffrischimpfung für besonders gesundheitlich gefährdete bzw. exponierte Personengruppen**
		**Nuvaxovid für ≥** **18-Jährige**
*19. Aktualisierung* [35]	31.03.2022	**COVID-19-Impfung mit mRNA-Impfstoffen von Personen, die mit einem der nicht in der EU zugelassenen Ganzvirusimpfstoffe **(CoronaVac, Covilo und Covaxin)** oder **dem Vektor-basierten Impfstoff **Sputnik V vorgeimpft sind**
*20. Aktualisierung* [20, 36]	25.05.2022; online vorab am 24.05.2022	**Generelle Impfempfehlung für 5‑ bis 11-jährige Kinder mit zunächst *einer* Impfstoffdosis**
		**Impfung von Personen, die eine gesicherte SARS-CoV-2-Infektion durchgemacht haben **(Aktualisierung)

Die schnelle Folge von Aktualisierungen der COVID-19-Impfempfehlungen war erforderlich, da neue Impfstoffe zugelassen wurden, bereits zugelassene Impfstoffe Indikationserweiterungen (z. B. neue Altersgruppen, Anwendung zur Auffrischimpfung, andere Dosierung für Personen mit Immundefizienz) erhielten, unerwünschte Wirkungen nach Impfungen auftraten, die eine Neubewertung der Risiko-Nutzen-Analyse erforderten (z. B. nach Auftreten thromboembolischer Ereignisse nach der Impfung mit Vaxzevria (AstraZeneca)), und sich die Epidemiologie von SARS-CoV‑2 kontinuierlich durch Auftreten neuer Virusvarianten mit sich änderndem Transmissionsverhalten und Krankheitsbild weiterentwickelte.

Einzelne Entscheidungen mussten rasch getroffen werden, ohne dass veröffentlichte Daten verfügbar waren, wie zum Beispiel die Empfehlung zur heterologen Impfung mit einem mRNA-Impfstoff nach Erstimpfung mit Vaxzevria bzw. JCOVDEN (Janssen Cilag International) nachdem die Indikationsgruppen für diese Impfstoffe nach Auftreten thromboembolischer Ereignisse von der STIKO geändert wurde. Hier war der Austausch mit Impfkommissionen anderer Länder, Arbeitsgruppen und ExpertInnen im In- und Ausland hilfreich (z. B. Vereinigtes Königreich, Israel, USA), die zum Teil noch unveröffentlichte Ergebnisse oder den Stand ihrer laufenden Forschung den STIKO-Mitgliedern in den Beratungen vorstellten.

Alle Aktualisierungen der STIKO-Empfehlungen sind vor der endgültigen Beschlussfassung als Beschlussentwürfe mit den dazugehörigen wissenschaftlichen Begründungen in ein beschleunigtes Stellungnahmeverfahren der betroffenen Fachkreise (medizinische Fachgesellschaften, Deutscher Ethikrat, Landesgesundheitsämter der Bundesländer, PEI, BMG, G‑BA) gegangen. In pandemiefreien Zeiten wird den Stellungnehmenden eine Frist von 6 Wochen zur Prüfung und Begutachtung eingeräumt. Während der Pandemie betrug die Rückmeldefrist in der Regel zwischen 24 h und 3 Arbeitstagen. Trotz der Kürze der Fristen haben die angefragten Fachkreise umfassend kommentiert und der STIKO so die Möglichkeit gegeben, ihre Empfehlungen unter Berücksichtigung der Rückmeldungen erneut zu diskutieren, ggf. anzupassen und somit auf breiten Konsens zu bauen. In den STIKO-Beratungen wurde intensiv diskutiert. Trotz der oft eingeschränkten Evidenzlage und daraus resultierenden unterschiedlichen Interpretationen wurden die meisten Entscheidungen einstimmig getroffen.

Die Publikationen der Aktualisierungen der COVID-19-Impfempfehlung im Epidemiologischen Bulletin wurden meist durch eine gleichzeitig veröffentlichte Pressemitteilung begleitet. Zusätzlich erfolgte eine Begleitkommunikation über STIKO-App-Push-Nachrichten, FAQs, verschiedene Faktenblätter zur COVID-19-Impfung und Interviews mit STIKO-Mitgliedern durch das Science Media Center. Alle Informationsmaterialen wurden unter Berücksichtigung der Aktualisierungen fortlaufend aktuell gehalten.

Im Anschluss an die COVID-19-Pandemie planen die Mitglieder der Kommission und die MitarbeiterInnen der STIKO-Geschäftsstelle sich für eine Evaluation zusammenzufinden und sich über die Erfahrungen in der Pandemie auszutauschen. Für die Bewältigung zukünftiger pandemischer Ereignisse ist es immens wichtig, *Lessons Learned* abzuleiten, auf die in Zukunft schnell zurückgegriffen werden kann.

Im Folgenden sollen anhand einzelner Beispiele die besonderen Herausforderungen der Kommissionsarbeit während der COVID-19-Pandemie dargestellt werden.

## Besondere Herausforderungen während der COVID-19-Pandemie

### Kontinuierliche Anpassung der Empfehlung aufgrund neuer Daten zu den COVID-19-Impfstoffen

Die Effektivitäts- und Sicherheitsdaten aus klinischen Impfstoffstudien bilden das Kernstück einer jeden Entscheidung für eine neue Impfempfehlung. Während der COVID-19-Pandemie lagen zwischen Zulassung und Einsatz der Impfstoffe in der Bevölkerung teilweise nur wenige Stunden bis Tage. Für die Erstellung ihrer Impfempfehlung lagen der STIKO daher nur die Effektivitäts- und Sicherheitsdaten aus den klinischen Impfstoffstudien vor, die zumeist erst kurz vor Impfstoffzulassung veröffentlicht wurden und, je nach Auswahl der Studienpopulationen, nur Teile der Bevölkerung abbildeten (z. B. bestimmte Altersgruppen). Es obliegt der STIKO, die verfügbare Evidenz kritisch zu bewerten, die Übertragbarkeit der Studiendaten auf die hiesige Bevölkerung zu prüfen und in einer abschließenden Nutzen-Risiko-Abwägung unter Einbezug weiterer Aspekte (z. B. Epidemiologie, Modellierungsergebnisse) zu entscheiden, ob und, wenn ja, für wen der jeweilige Impfstoff empfohlen werden sollte.

So wurde beispielsweise der Vektor-basierte Impfstoff Vaxzevria, den die EMA am 29.01.2021 für alle Altersgruppen ≥ 18 Jahre zugelassen hatte (Abb. 1), aufgrund fehlender Wirksamkeitsdaten für die ältere Bevölkerung von der STIKO zunächst nur für 18- bis 64-Jährige empfohlen [7].

Die STIKO hat bei ihren Entscheidungen über die Empfehlungen zur COVID-19-Impfung die Ergebnisse aus der infektionsdynamischen Modellierung genutzt, wenn sich die Frage stellte, ob die Impfung für eine neue bzw. weitere Altersgruppe empfohlen werden sollte. Dabei war es wichtig zu prüfen, ob durch die Impfung Effekte auf Bevölkerungsebene zu erwarten wären, die das COVID-19-Infektionsgeschehen positiv beeinflussen würden. Es wurde differenziert zwischen direkten Effekten (verhinderte SARS-CoV-2-Infektionen und COVID-19-Fälle in der neu geimpften Bevölkerungsgruppe) und indirekten Effekten (durch reduzierte Transmission verhinderte SARS-CoV-2-Infektionen und COVID-19-Fälle in anderen Altersgruppen). Auf Basis einer angenommenen Impfquote und der aktuellen SARS-CoV-2-Infektionslage wurden diese Effekte modelliert und so eine Nutzen-Risiko-Abwägung ermöglicht. Auf der Nutzenseite wurden die positiven direkten und indirekten Effekte abgeschätzt. Diese Ergebnisse wurden den Analysen der Risikoseite (die Zahl der zu erwartenden schweren unerwünschten Impfreaktionen, z. B. Myokarditiden (Herzmuskelentzündungen)) gegenübergestellt. Die Modellierergebnisse haben wertvolle Informationen für die abschließende Entscheidung geliefert. Keinen zusätzlichen Nutzen hatte das Modell bei Entscheidungen über die Einführung einer Empfehlung in bestimmten gefährdeten Bevölkerungsgruppen, deren Größe so gering war, dass durch den Impfschutz in dieser Gruppe weder Auswirkungen auf den Pandemieverlauf noch Effekte auf Bevölkerungsebene zu erwarten waren. Hier sei beispielhaft die COVID-19-Impfempfehlung für Schwangere und für Immundefiziente genannt.

Nach der Impfstoffzulassung werden aufgrund des Einsatzes in großen Teilen der Weltbevölkerung fortlaufend neue Daten zur Effektivität und Sicherheit der Impfstoffe in klinischen Studien, Beobachtungsstudien, Fallserien etc. unter *Real-live*-Bedingungen erhoben. Anhand dieser Daten überprüft die STIKO ihre bestehenden Empfehlungen kontinuierlich und passt diese zeitnah, wenn nötig, an, um durch die Impfungen stets den bestmöglichen Nutzen für die Bevölkerung zu erreichen und Risiken zu minimieren. Beispielhaft sei hier die Entscheidungsserie der STIKO zum Impfstoff Vaxzevria aufgeführt: Der Impfstoff Vaxzevria war, wie oben bereits erwähnt, Ende Januar 2021 aufgrund fehlender Wirksamkeitsdaten für die ältere Bevölkerung zunächst nur für 18- bis 64-Jährige von der STIKO empfohlen worden [7]. Von anderen Impfstoffen ist bekannt, dass die Wirksamkeit im höheren Alter eingeschränkt sein kann. Nach Veröffentlichung zweier großer Beobachtungsstudien aus England und Schottland [8, 9], die eine ausreichende Effektivität auch bei ≥ 70-Jährigen nachwiesen, wurde die Impfempfehlung nach 6 Wochen Mitte März 2021 auf die Altersgruppe ≥ 18 Jahre ausgeweitet. Nachdem etwa gleichzeitig ein Sicherheitssignal bei der Anwendung von Vaxzevria bei < 60-Jährigen festgestellt wurde, passte die STIKO ihre Empfehlung erneut an und sprach Anfang April diese ausschließlich für ≥ 60-Jährige aus. In der Öffentlichkeit wurde dieser notwendige, der wissenschaftlichen Praxis entsprechende Anpassungsmechanismus teilweise als Ausdruck von Unentschiedenheit der STIKO wahrgenommen. Der geschilderte Entscheidungsprozess macht jedoch deutlich, dass die STIKO evidenzbasiert und zugleich sehr schnell auf eine veränderte Studienlage reagiert hat.

#### Anpassung der Empfehlung für Kinder und Jugendliche

Nach Zulassung des mRNA-Impfstoffs Comirnaty für Kinder und Jugendliche im Alter von 12 bis 17 Jahren hat die STIKO im Juni 2021 nach abschließender Risiko-Nutzen-Abwägung entschieden, zunächst keine allgemeine COVID-19-Impfempfehlung für diese Altersgruppe auszusprechen. Sie hat vielmehr eine Indikationsimpfempfehlung für Kinder und Jugendliche mit Vorerkrankungen und mit engem Kontakt zu vulnerablen Personen, die selbst nicht ausreichend durch eine Impfung geschützt werden können, gegeben. Ausschlaggebend war trotz der hohen Impfeffektivität zum Schutz vor COVID-19 vor allem die Einschätzung der STIKO, dass hinsichtlich der Sicherheit des Impfstoffes, insbesondere des Risikos einer Myokarditis nach Impfung, noch Wissenslücken bestanden. Nach Einschätzung der STIKO war zudem der Nutzen der Impfung hinsichtlich der Verhinderung schwerer Erkrankungen und Todesfälle in dieser Altersgruppe nicht allgemein gegeben, da Hospitalisierungen und intensivmedizinische Behandlungen aufgrund von COVID-19 bei Kindern und Jugendlichen selten sind. Die Studienlage zum Schutz der Impfung vor dem *Pediatric Inflammatory Multisystem Syndrome* (PIMS) und vor Long-COVID war uneinheitlich und ließ keine definitive Einschätzung zu. Im Juni 2021 gab es zudem in Deutschland noch einen Impfstoffmangel und nicht ausreichende Impfkapazitäten. Die STIKO hat daher Wert darauf gelegt, dass die limitierten Ressourcen nutzbringend eingesetzt werden und vorrangig noch nicht geimpften gefährdeten Personen ein Impfangebot gemacht werden sollte. Solange noch viele ältere Erwachsene mit deutlich höherem Risiko ungeimpft waren, war eine Umverteilung der Impfstoffe an gesunde Kinder und Jugendliche epidemiologisch und individualmedizinisch nicht sinnvoll.

Die STIKO hat bereits im August 2021 eine erneute Nutzen-Risiko-Abwägung vorgenommen, als Daten zum Auftreten von Myokarditiden aus der Anwendung der Impfstoffe bei 12- bis 17-Jährigen in den USA und Kanada vorlagen. Bei verbesserter Impfstoffverfügbarkeit und einer sich ändernden Epidemiologie durch das Auftreten der bedrohlichen Delta-Variante hat die STIKO eine allgemeine COVID-19-Impfempfehlung für 12- bis 17-Jährige ausgesprochen.

Im Dezember 2021 wurde dann auch ein Impfstoff in geringerer Dosierung für die Altersgruppe der 5‑ bis 11-Jährigen zugelassen. Bei erneut mangelhafter Datenlage zur Impfstoffsicherheit und Unklarheit hinsichtlich des Myokarditisrisikos hat die STIKO auch für die jüngeren Kinder zunächst keine allgemeine Impfempfehlung ausgesprochen. Im Mai 2022, unter der durch unterschiedliche Omikron-Varianten geprägten Epidemiologie, hat die STIKO ihre COVID-19-Impfempfehlung für 5‑ bis 11-Jährige erneut aktualisiert. Auf Basis der Meldedaten wurde geschätzt, dass zu diesem Zeitpunkt > 80 % der 5‑ bis 11-Jährigen bereits eine SARS-CoV-2-Infektion durchgemacht hatten. Sicherheitsdaten aus der breiten Anwendung in anderen Ländern hatten inzwischen gezeigt, dass die Impfung gut verträglich und das Myokarditisrisiko nach Impfung in dieser Altersgruppe sehr gering ist. Gleichzeitig waren die beobachteten Krankheitsverläufe durch die Omikron-Variante überwiegend mild und die Schutzwirkung der Impfung gegenüber Omikron-Infektionen schon wenige Monate nach der Impfung deutlich reduziert. Zahlreiche Studien zeigten inzwischen, dass erwachsene Personen, die eine Infektion durchgemacht haben und mindestens eine Impfstoffdosis erhalten hatten, also eine hybride Immunität aufwiesen, am besten vor schweren Verläufen geschützt waren. Für Kinder lagen solche Untersuchungen bisher nicht vor. Ein erneuter Anstieg von SARS-CoV-2-Infektionen im Herbst bzw. Winter 2022 wird erwartet. Es ist nicht absehbar, welche Varianten dann vorherrschen werden. Die STIKO hat daher entschieden, nun auch gesunden Kindern zwischen 5–11 Jahren *zunächst eine* Impfstoffdosis gegen COVID-19 zu empfehlen. Diese Impfempfehlung wurde vorsorglich ausgesprochen und zielt darauf ab, durch Erreichung einer hybriden Immunität eine möglichst gute Basisimmunität aufzubauen.

### Impfnebenwirkungen (schwere unerwünschte Ereignisse) machen eine kurzfristige Anpassung der Impfempfehlung notwendig

Die umgehende Reaktion auf ernsthafte Impfzwischenfälle ist wichtig, um individuellen Schaden zu begrenzen und durch ein überlegtes, rasches Handeln das Vertrauen in die Impfung, die zuständigen staatlichen Stellen und die Impfkommission zu stärken.

In Deutschland ist das Paul-Ehrlich-Institut (PEI) für die Beurteilung der Sicherheit von Impfstoffen verantwortlich. Über das seit langer Zeit etablierte Meldesystem werden Verdachtsfälle von Impfnebenwirkungen gemeldet und erfasst. So können zeitnah Signale, wie z. B. Myokarditiden nach mRNA-Impfstoffen, detektiert und das Nutzen-Risiko-Profil der Impfstoffe kontinuierlich überwacht werden. Die STIKO berücksichtigt die Ergebnisse der Sicherheitsevaluation und passt ihre Impfempfehlungen ggf. entsprechend an. Im Folgenden möchten wir 2 Situationen darstellen, die zu einer Risiko-basierten Änderung der COVID-19-Impfempfehlung geführt haben. Diese Ereignisse belegen eindrucksvoll, welche Bedeutung der zeitnahen Meldung, Analyse und Bewertung von schweren unerwünschten Impfnebenwirkungen zukommt.

#### Einschränkung der Indikation für Vaxzevria und JCOVDEN

Die Empfehlungen zur Zulassung der Vektor-basierten Impfstoffe Vaxzevria und JCOVDEN wurden im Winter bzw. Frühjahr 2021 von der EMA gegeben (Abb. 1). Nach Zulassung der Impfstoffe durch die EU hat die STIKO die Impfung mit Vaxzevria ab Ende Januar 2021 [7] und den Impfstoff JCOVDEN ab Mitte April 2021 [10] in ihren Empfehlungen berücksichtigt. Nach der Impfung mit Vaxzevria sind in Deutschland und in anderen Ländern Anfang März 2021 mehrere Fälle von Sinusvenenthrombosen (SVT) in Kombination mit Thrombozytopenien (Mangel an Blutplättchen) bei Geimpften mit z. T. tödlichem Ausgang beobachtet worden (Thrombose-mit-Thrombozytopenie-Syndrom; TTS; [11]). Die Symptome des potenziell lebensbedrohlichen Krankheitsbildes traten 4–16 Tage nach der Verabreichung der Impfung auf [10]. Bis zum 31.03.2021 berichtete das PEI aus der Spontanerfassung unerwünschter Wirkungen über insgesamt 31 Frauen und 7 Männer mit einer SVT, die zwischen dem 13.02.2021 und dem 23.03.2021 mit Vaxzevria immunisiert worden waren. Bis auf 2 Fälle bei Frauen waren alle betroffenen Personen 20–55 Jahre alt. 7 PatientInnen verstarben.

Auf Basis der vorliegenden Daten führte das PEI unter Berücksichtigung der Hintergrundinzidenz für SVT und der Impfquoten eine Observed-versus-Expected-Analyse durch und stellte fest, dass die Zahl der gemeldeten („observed“) SVT bei Frauen im Alter von 18 bis 59 Jahren deutlich höher als die im Zeitintervall von 16 Tagen zu erwartende („expected“) Anzahl von SVT war. Es wurde ermittelt, dass im Zeitraum von 16 Tagen nach Verabreichung der Impfung bei 20- bis 59-Jährigen mit dem Auftreten von zusätzlich 2–3 SVT/100.000 geimpfte Frauen und 1–2 SVT/100.000 geimpfte Männer gerechnet werden muss [10].

Unter Berücksichtigung dieser Ergebnisse führte die STIKO eine altersabhängige Risiko-Nutzen-Bewertung hinsichtlich der Anwendung von Vaxzevria durch und entschied innerhalb kürzester Zeit die Impfung mit Vaxzevria nur noch für Personen im Alter von ≥ 60 Jahren zu empfehlen. Denn anders als in der Altersgruppe der < 60-Jährigen ist der Nutzen der Impfung bei den > 60-Jährigen aufgrund des hohen Risikos schwerer COVID-19-Verläufe sehr hoch und gleichzeitig ihr Risiko für thromboembolischen Ereignisse sehr niedrig (95 % der gemeldeten thromboembolischen Ereignisse traten bei < 60-Jährigen auf). Personen < 60 Jahre, die bereits eine 1. Dosis Vaxzevria erhalten hatten, wurde in der Folge die Gabe von einer mRNA-Impfstoffdosis empfohlen (sog. heterologes Impfschema; [12]).

Nachdem in den USA auch nach Anwendung von JCOVDEN Fälle von TTS überwiegend bei jüngeren Geimpften aufgetreten waren, beschloss die STIKO am 12.05.2022, die Vektor-basierten Impfstoffe Vaxzevria und JCOVDEN generell nur für Menschen im Alter ≥ 60 Jahren zu empfehlen [12]. Diese Empfehlung wurde vorsorglich getroffen. In Deutschland waren zu diesem Zeitpunkt noch keine Fälle von TTS nach JCOVDEN-Impfung beobachtet worden.

#### Einschränkung der Indikation für Spikevax

Die mRNA-Impfstoffe Comirnaty und Spikevax (Moderna) wurden im Dezember 2020 bzw. Januar 2021 als erste COVID-19-Impfstoffe von der EU zugelassen. Im Juli 2021 stellte der Ausschuss für Risikobewertung im Bereich der Pharmakovigilanz (PRAC) der EMA einen möglichen kausalen Zusammenhang zwischen mRNA-Impfstoffen und dem Auftreten von Myo- und Perikarditiden (Herzmuskel- und Herzbeutelentzündungen) fest [13]. Die Symptomatik der Myo- und Perikarditiden trat innerhalb von wenigen Tagen nach der Impfung auf und die Fälle waren nach der 2. Impfstoffdosis häufiger als nach der 1. Dosis. Myo- und Perikarditiden wurden bei Jungen und jungen Männern (< 30 Jahren) deutlich häufiger beobachtet als bei Mädchen und jungen Frauen desselben Alters sowie allgemein bei ≥ 30-Jährigen [14]. Nach Informationen aus dem Sicherheitsbericht gingen bis zum 30.09.2021 im PEI 1243 Verdachtsmeldungen einer Myo- und Perikarditis nach mRNA-Impfung ein [15]. Um das Sicherheitssignal abzuklären, führte das PEI im Rahmen der Pharmakovigilanz eine Observed-versus-Expected-Analyse durch und prüfte, ob die Anzahl der beobachteten Myokarditisfälle über der Hintergrundinzidenz in der Bevölkerung lag [16]. Die Daten der Spontanerfassung zu Myo- und Perikarditiden nach mRNA-Impfung in Deutschland deuteten darauf hin, dass das Risiko nach Gabe von Spikevax bei Jungen und jungen Männern (< 30 Jahren) sowie bei Mädchen und jungen Frauen < 30 Jahren deutlich höher ist als nach Gabe von Comirnaty. Internationale Berichte zum Auftreten von Myo- und Perikarditiden nach der Verabreichung von mRNA-Impfstoffen bestätigten diese Ergebnisse.

Die STIKO führte eine Risiko-Nutzen-Analyse durch und entschloss sich, obwohl die Fälle insgesamt selten sind, ihre Impfempfehlung anzupassen und Spikevax nur noch für ≥ 30-Jährige zu empfehlen. Personen im Alter < 30 Jahren sollten einen anderen Impfstoff erhalten.

### Dynamik der Pandemie, der Impfstoffentwicklung und der Evidenzgenerierung

Neben der andauernden Weiterentwicklung der Evidenzlage in Bezug auf die Effektivität und Sicherheit der COVID-19-Impfstoffe erwächst eine besondere Komplexität bei der Erarbeitung der Impfempfehlungen aus der sich stetig ändernden Epidemiologie von SARS-CoV‑2 (Krankheitsschwere, Transmissionsverhalten, unterschiedliche Formen des Immunescape). Diese resultiert aus den in kurzer Folge neu auftretenden Virusvarianten und der sich gleichzeitig ändernden Immunitätslage in der Bevölkerung, sei es durch durchgeführte Impfungen und/oder durchgemachte Infektionen. Um beispielsweise im Dezember 2021 auf die sich rasch ausbreitende Omikron-Variante zu reagieren, die nachweislich einen ausgeprägteren Immunescape zeigte als vorangegangene Varianten, wurde das Impfintervall für Auffrischimpfungen von zunächst 6 auf 3 Monate verkürzt, um die Immunität in der Bevölkerung rasch zu erhöhen.

Auch wurde die Empfehlung für eine Impfung von Personen, die eine SARS-CoV-2-Infektion durchgemacht haben, mehrfach aufgrund von neuen Erkenntnissen angepasst. Im Frühjahr 2021 empfahl die STIKO zunächst keine Impfung nach durchgemachter Infektion. Dies geschah in Unkenntnis der Schutzdauer nach durchgemachter Infektion, jedoch in der Annahme eines Schutzes vor erneuter Infektion für mehrere Monate, basierend auf Erfahrungen mit anderen Viruserkrankungen und angesichts des bestehenden Impfstoffmangels [5]. Im Juli 2021, unter Dominanz der Delta-Variante, erfolgte die Empfehlung einer einzelnen Impfstoffdosis im Abstand von 6 Monaten zur Infektion [17]. Ende November 2021 wurde eine weitere Impfung als Auffrischimpfung im Abstand von erneut 6 Monaten für Personen empfohlen, die eine SARS-CoV-2-Infektion durchgemacht hatten [18]. Im Dezember wurde unter der beginnenden Zirkulation der Omikron-Variante der Impfabstand nach Infektion und für die Auffrischimpfung jeweils von 6 auf 3 Monate verkürzt [19]. Eine erneute Aktualisierung der Empfehlung für Personen, die eine SARS-CoV-2-Infektion durchgemacht haben, wurde aufgrund von aktuellen Daten zur hybriden Immunität im Mai 2022 verabschiedet. Die Daten zeigen, dass die Immunantwort nach 3 Antigenkontakten (Kombination von durchgemachten SARS-CoV-2-Infektionen und Impfungen) stärker und breiter ausfällt als nach alleiniger Infektion oder alleiniger Impfung. Die STIKO empfiehlt daher, dass auch Personen, die mehrere Infektionen durchgemacht haben, mindestens einmal geimpft werden sollen [20].

## Ausblick

In der COVID-19-Pandemie hat die STIKO fortlaufend unter Berücksichtigung der vorhandenen Daten evidenzbasierte Impfempfehlungen erstellt. Aufgrund ihrer Struktur als ehrenamtliche, nicht weisungsgebundene wissenschaftliche Expertenkommission und der gut etablierten Arbeitsweise zur Entwicklung evidenzbasierter Impfempfehlungen konnte die STIKO sich trotz des hohen Zeitdrucks und des großen öffentlichen Interesses ihre Unabhängigkeit bewahren.

Die Evidenzbasierung von Impfempfehlungen ist sowohl in „normalen“ Zeiten als auch während einer Pandemie notwendig, um eine breite Zustimmung in der Ärzteschaft und der Bevölkerung zu erzielen. Die Erarbeitung evidenzbasierter Empfehlungen benötigt Sorgfalt und Zeit. Es ist unerlässlich, der Kommission diese Zeit einzuräumen.

Nach dem Auftreten eines neuen Erregers gibt es eine Vielzahl neuer Erkenntnisse, die sich dynamisch verändern und kontinuierlich erweitern. Die Umstände sind oftmals komplex. Es hat sich während der Pandemie gezeigt, dass diese Zusammenhänge von Teilen der Öffentlichkeit oftmals schwer verstanden werden. Die STIKO hat ihre Kommunikationsstrategie ausgeweitet, angepasst und neue Formate der Begleitkommunikation aufgenommen, wie die Teilnahme an einordnenden Fachgesprächen, die durch das Science Media Center organisiert werden. Das Publikationsformat der STIKO-Impfempfehlungen und ihrer wissenschaftlichen Begründungen richtet sich in erster Linie an die impfende Fachöffentlichkeit. Im direkten Kontakt mit der Bevölkerung ist die impfende Ärzteschaft ein wichtiges Sprachrohr für Impfempfehlungen. Es bleibt jedoch die Aufgabe anderer Stellen, die Impfkampagne kommunikativ zu begleiten. Für die erfolgreiche Umsetzung von Impfempfehlungen ist neben der Kommunikation mit der Fachöffentlichkeit eine bevölkerungsorientierte Impfkampagne notwendig.

Die zurückliegenden Monate haben gezeigt, wie wichtig eine gute Ausstattung der STIKO-Geschäftsstelle und des Modellierungsteams im Fachgebiet Impfprävention am RKI mit weiteren personellen Ressourcen ist. Aktuell wurde ein Modellierer-Team mit 3 festen Stellen im Fachgebiet Impfprävention des RKI etabliert, welches der STIKO für den Entscheidungsprozess und die Nutzen-Risiko-Abwägung notwendige Modellierergebnisse zur Verfügung stellen soll. Auch die Geschäftsstelle der STIKO wurde personell mit 2 Wissenschaftlerinnen und einer Verwaltungsangestellten verstärkt. Für den Fall einer erneuten pandemischen Lage ist es aus Sicht der Geschäftsstelle jedoch notwendig, dass der personelle Bedarf rechtzeitig evaluiert wird. Die Verkürzung der Zeitintervalle bis zu Veröffentlichung einer Empfehlung wurde unter den aktuellen Pandemiebedingungen nicht durch eine Minimierung an Sorgfalt oder Vernachlässigung der Prinzipien der evidenzbasierten Medizin, sondern durch einen Mehraufwand an Arbeit bei allen Beteiligten erreicht. Da es sich gezeigt hat, dass ein erhöhter Kommunikationsbedarf während einer Pandemie besteht, sollte bei zukünftigen gesundheitlichen Notlagen frühzeitig eine umfassende Pressearbeit der STIKO gewährleistet werden.
